# Supplementary material for: MetaRibo-Seq measures translation in microbiomes
Source: Nat Commun. 2020 Jun 29;11:3268. doi: 10.1038/s41467-020-17081-z (PMC7324362; doi:10.1038/s41467-020-17081-z)
Supplement: Supplementary file 10 — Supplementary Data 7 [file 41467_2020_17081_MOESM10_ESM.zip › File2/Confidence_High_Taxonomy/178277_out.krona.html]

Javascript must be enabled to view this page.

members
magnitude
magnitudeUnassigned
count
unassigned
taxon
rank

178277\_out

8

superkingdom
8
2

phylum
8
976

200643
class
8

171549
order
8

1853231
8
family

283168
genus
8

28118

SRS011134\_contig\_number\_contig-100\_2682.246836SRS014412\_contig\_number\_11785SRS022609\_contig\_number\_44172SRS049773\_contig\_number\_contig-100\_722.207064SRS077194\_contig\_number\_contig-100\_1072.157460SRS1041129\_contig\_number\_contig-100\_2983.76323SRS1055022\_contig\_number\_contig-100\_1562.114087SRS893279\_contig\_number\_contig-100\_1523.95515
species
8
